# Growth hormone action predicts age-related white adipose tissue dysfunction and senescent cell burden in mice

**DOI:** 10.18632/aging.100681

**Published:** 2014-07-20

**Authors:** Michael B. Stout, Tamara Tchkonia, Tamar Pirtskhalava, Allyson K. Palmer, Edward O. List, Darlene E. Berryman, Ellen R. Lubbers, Carlos Escande, Adam Spong, Michal M. Masternak, Ann L. Oberg, Nathan K. LeBrasseur, Richard A. Miller, John J. Kopchick, Andrzej Bartke, James L. Kirkland

**Affiliations:** ^1^Robert and Arlene Kogod Center on Aging, Mayo Clinic, Rochester, MN 55905, USA; ^2^Edison Biotechnology Institute and Heritage College of Osteopathic Medicine, Ohio University, Athens, OH 45701, USA; ^3^Department of Internal Medicine, Southern Illinois University School of Medicine, Springfield, IL 62794, USA; ^4^Burnett School of Biomedical Sciences, University of Central Florida, Orlando, FL 32827, USA; ^5^Division of Biomedical Statistics and Informatics, Department of Health Sciences Research, Mayo Clinic, Rochester, MN 55905, USA; ^6^Department of Pathology and Geriatrics Center, University of Michigan, Ann Arbor, MI 48109, USA

**Keywords:** adipose tissue, aging, cellular senescence, growth hormone

## Abstract

The aging process is associated with the development of several chronic diseases. White adipose tissue (WAT) may play a central role in age-related disease onset and progression due to declines in adipogenesis with advancing age. Recent reports indicate that the accumulation of senescent progenitor cells may be involved in age-related WAT dysfunction. Growth hormone (GH) action has profound effects on adiposity and metabolism and is known to influence lifespan. In the present study we tested the hypothesis that GH activity would predict age-related WAT dysfunction and accumulation of senescent cells. We found that long-lived GH-deficient and -resistant mice have reduced age-related lipid redistribution. Primary preadipocytes from GH-resistant mice also were found to have greater differentiation capacity at 20 months of age when compared to controls. GH activity was also found to be positively associated with senescent cell accumulation in WAT. Our results demonstrate an association between GH activity, age-related WAT dysfunction, and WAT senescent cell accumulation in mice. Further studies are needed to determine if GH is directly inducing cellular senescence in WAT or if GH actions on other target organs or alternative downstream alterations in insulin-like growth factor-1, insulin or glucose levels are responsible.

## INTRODUCTION

Growth hormone (GH) plays a central role in regulating mammalian growth, metabolic homeostasis, and adiposity. GH, either independently or through insulin-like growth factor-1 (IGF-1), has profound effects on age-related disease onset and longevity. Both mice and humans with excessive GH production have increased mortality rates compared to age-matched controls [1, 2], presumably due to higher incidence of metabolic dysfunction and cancer. Conversely, decreased GH activity is associated with dramatic lifespan extension in mice and similar effects in rats [3-7]. The effect of diminished GH activity on human lifespan is yet to be definitively elucidated, although these populations do appear to be protected from age-related metabolic dysfunction and cancer [8]. Furthermore, low levels of circulating IGF-1 predicts exceptional longevity in humans [9]. Several different GH-related gene mutations elicit lifespan extension in mice, regardless of genetic background or diet administered [10].

Ames and Snell dwarf mice were the first GH-deficient genetic mutants to display an extension of lifespan [5, 6]. Ames dwarf mice are homozygous for a recessive mutation in the prophet of pituitary transcription factor-1 (*prop-1*) gene [11]. Prop-1 induces the expression of pituitary transcription factor-1 (Pit-1), which is needed for pituitary cells to acquire capacity to produce GH, prolactin (PRL), and thyroid-stimulating hormone (TSH) [12]. Hence, Ames dwarf mice are deficient in all three hormones. Ames dwarfs live approximately 50% longer than non-mutant siblings and are protected from age-related metabolic perturbations and cancer [10, 13]. Snell dwarf mice are homozygous for a recessive mutation in the Pit-1 gene (*pou1f1*) [12]. As seen in Ames dwarfs, Snell dwarf mice are deficient in GH, PRL, and TSH, have increased longevity, and delayed onset of metabolic disorders and cancer as they age. The Snell dwarfs live approximately 40% longer than non-mutant littermates. [6, 10, 13]. Confirming these findings in Ames and Snell dwarfs without confounding deficiencies in PRL and TSH, GH-resistant mutant mice display similar phenotypic characteristics [7]. Mice with homozygous deletion of the GH receptor (GHR-/-) are dwarfs and have elevated levels of circulating GH, likely due to severely decreased circulating IGF-1 and inhibition of the long hypothalamic negative feedback loop. As in Ames and Snell mutants, GHR-/- mice are long-lived and protected from age-related metabolic dysfunction and tumor burden [10, 13]. GHR-/- mice live approximately 30% longer than wild-type littermates independent of genetic background [7, 14].

In contrast to the GH-deficient and -resistant mutants, mice with increased GH activity display characteristics resembling premature aging. Transgenic bovine GH-overexpressing (bGH) mice are large, have high levels of circulating IGF-1, develop metabolic abnormalities early in life, have increased cancer incidence, and lifespans that are approximately 30% shorter than wild-type mice [1, 15]. Interestingly, the degree of GH activity is inversely associated with age-related insulin sensitivity and systemic inflammation in all the aforementioned mutants [10, 13]. Collectively, these findings suggest links between aging processes and inflammatory and metabolic pathways in GH-responsive organ systems.

Mammalian aging is associated with profound changes in lipid deposition and low-grade tissue inflammation. These phenotypes correspond to declines in subcutaneous white adipose tissue (WAT) function and increased ectopic lipid accumulation [16-18]. Among the mechanisms that contribute to age-related decreases in WAT lipid storage are decreased preadipocyte replication [19-22] and adipogenic potential [21, 23, 24]. Inflammation is likely involved, with inflammatory mediators contributing to inhibited preadipocyte differentiation [23-25]. The source of inflammation is still unclear, although preadipocytes have been shown to increase pro-inflammatory cytokine, chemokine, and extracellular matrix protease expression with aging [26, 27]. Additionally, the accumulation of senescent cells has emerged as a feature of aging WAT [28]. Senescent cells typically have a pro-inflammatory secretory profile, termed the senescence-associated secretory phenotype (SASP) [29-32], which can adversely affect the local microenvironment through tissue remodeling and apoptosis [32]. Removal of senescent cells partly restores WAT mass and improves healthspan in mice with progeroid syndrome [33]. Collectively, these findings suggest senescent cells may play a role in WAT dysfunction with chronological aging. Based on the connections among GH activity, metabolic homeostasis, systemic inflammation, and longevity, we tested the hypothesis that GH action can predict age-related WAT dysfunction and accumulation of senescent cells.

## RESULTS

### Age-related lipid redistribution is blunted with diminished GH activity

**Figure 1:**
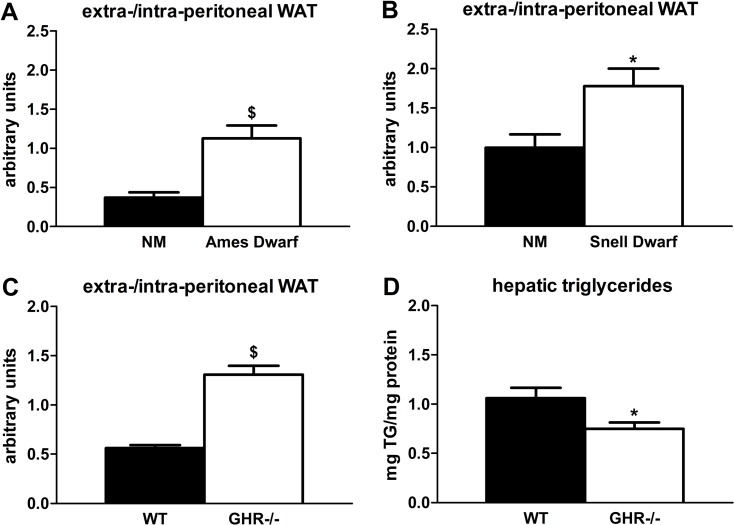
Age-related lipid redistribution is decreased in long-lived mice with diminished GH activity. (**A**) Ratios of extra-/intra-peritoneal (visceral) WAT in 18-month old female Ames dwarf and age-matched non-mutant (NM) littermates. (**B**) Ratios of extra-/intra-peritoneal WAT in 18-month old female Snell dwarf and age-matched non-mutant (NM) littermates. (**C**) Ratios of extra-/intra-peritoneal WAT in 18-month old female GHR-/- and age-matched wild-type (WT) littermates. (**D**) Hepatic triglyceride content in 18-month old female GHR-/- and age-matched wild-type (WT) littermates. Data were analyzed by Student's t-test and are expressed as mean ± SEM of 6 mice per group for A-B, 5 mice per group for C, and 8 mice per group for D. ^*^P<0.05; ^$^P<0.005.

Lipid redistribution frequently occurs in aging mammals [28], so delayed aging phenotypes could be associated with reduced lipid redistribution. Indeed, we found that GH-deficient and -resistant mutants have preservation of extra-peritoneal WAT at 18 months of age. Ames dwarf mice had more than 3-fold higher extra-/intra-peritoneal WAT ratios compared to age-matched non-mutant littermates (P=0.002; Figure 1A), which supports a previous report in which Ames dwarfs had reduced epididymal WAT triglyceride accumulation [34]. Snell dwarf mice were similar: they had a nearly 2-fold higher extra-/intra-peritoneal WAT ratio compared to age-matched non-mutant littermates (P=0.019; Figure 1B). The GHR-/- mice also had increased extra-/intra-peritoneal WAT ratios (2.3-fold; P<0.001) *vs*. wild-type littermates, consistent with reports by Berryman et al. in which GHR-/- mice had more subcutaneous WAT than controls throughout the life cycle [35, 36]. Additional evidence for reduced lipid redistribution is our finding that GHR-/- mice accumulated 30% less hepatic triglycerides than wild-type littermates (P=0.028), although a previous study did not demonstrate statistically significant differences in hepatic triglyceride content between GHR-/- and wild-type littermates [36].

### Age-related declines in preadipocyte differentiation are blunted in GH-resistant mice

**Figure 2:**
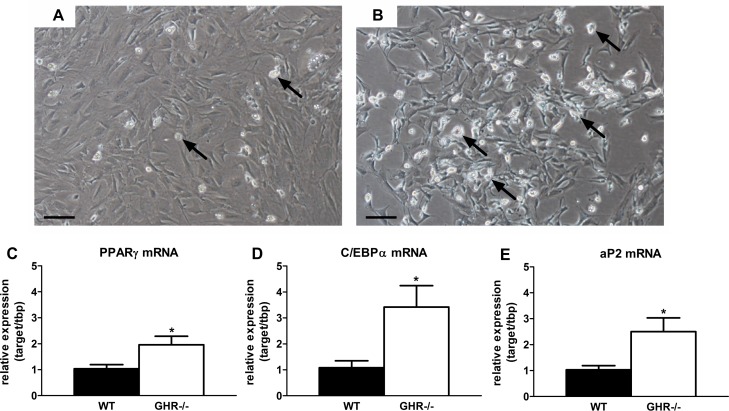
Preadipocyte differentiation is preserved in long-lived GHR-/- mice. (**A**) Representative image of ING preadipocyte differentiation from 20-month old female wild-type mice following exposure to differentiation media for 48 hours. (**B**) Representative image of ING preadipocyte differentiation from 20-month old female GHR-/- mice following exposure to differentiation media for 48 hours. (**C**) PPARγ mRNA expression in differentiating ING preadipocytes from female GHR-/- and age-matched wild-type (WT) littermates. (**D**) C/EBPα mRNA expression in differentiating ING preadipocytes from female GHR-/- and age-matched wild-type (WT) littermates. E. aP2 mRNA expression in differentiating ING preadipocytes from female GHR-/- and age-matched wild-type (WT) littermates. Data were analyzed by Student's t-test and are expressed as mean ± SEM of 4 mice per group. Scale Bar: A-B=100μm; Arrows indicate differentiating cells, which contain doubly bi-refractile lipid droplets.^*^P<0.05.

Preadipocyte differentiation capacity is reduced with increasing age in rodents and humans [28]. We surmised that preservation of extra-peritoneal WAT in mice with blunted GH activity may be related to increased differentiation. We found that primary preadipocytes isolated from inguinal (ING) WAT in GHR-/- mice (20 months old) accumulated more lipid than wild-type siblings after exposure to differentiation media for 48 hours (Figure 2A-B). Expression of key adipogenic transcriptional markers was higher in preadipocytes cultured from GHR-/- than wild-type mice. Peroxisome proliferator-activated receptor gamma (PPARγ) expression was nearly 2-fold higher in differentiating preadipocytes isolated from GHR-/- mice than wild-type littermates (P=0.044; Figure 2C). CCAAT/enhancer-binding protein alpha (C/EBPα) expression was also 3-fold higher in cells from GHR-/- mice than littermate controls (P=0.036; Figure 2D). Adipocyte protein 2 (aP2, or FABP4) expression, a differentiation-dependent gene target of C/EBPα and PPARγ, was increased over 2-fold in differentiating preadipocytes from GHR-/- *vs*. wild-type mice (P=0.038; Figure 2E).

### Age-related senescent cell burden in WAT is associated with GH activity

**Figure 3:**
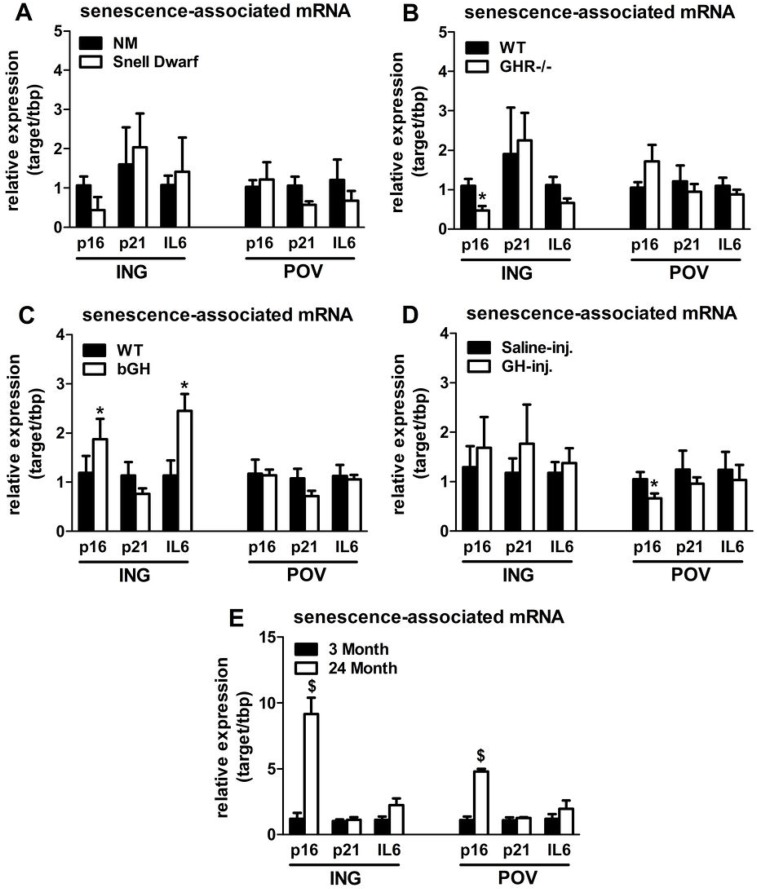
Lifelong GH activity predicts senescence-associated gene expression in WAT. (**A**) Expression of p16, p21, and IL6 in WAT from 18-month old female Snell dwarf and age-matched non-mutant (NM) littermates. (**B**) Expression of p16, p21, and IL6 in WAT from 18-month old female GHR-/- and age-matched wild-type (WT) littermates. C. Expression of p16, p21, and IL6 in WAT from 10-month old female bGH and age-matched wild-type (WT) controls. D. Expression of p16, p21, and IL6 in WAT from 19-month old female GH-injected (GH-inj.) and age-matched saline-injected (Saline-inj.) controls. E. Expression of p16, p21, and IL6 in WAT from female 24 month and 3 month old mice. Data were analyzed by Student's t-test and are expressed as mean ± SEM of 4 mice per group for A & E and 6 mice per group for B-D. ^*^P<0.05; ^$^P<0.005.

**Figure 4:**
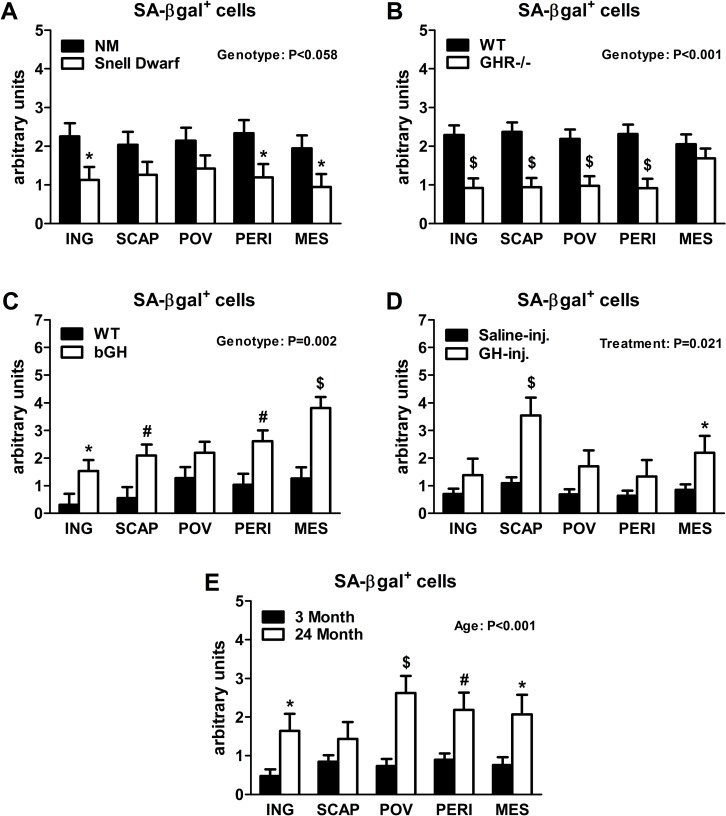
Lifelong GH activity predicts senescent cell accumulation in WAT. (**A**) SA-βgal^+^ cells in 18-month old female Snell dwarf and age-matched non-mutant (NM) littermates. (**B**) SA-βgal^+^ cells in 18-month old female GHR-/- and age-matched wild-type (WT) littermates. (**C**) SA-βgal^+^ cells in 10-month old female bGH and age-matched wild-type (WT) controls. (**D**) SA-βgal^+^ cells in 19-month old female GH-injected (GH-inj.) and age-matched saline-injected (Saline-inj.) controls. E. SA-βgal^+^ cells in female 24 month and 3 month old mice. SA-βgal^+^ data were analyzed by a mixed effects model. All data are expressed as mean ± SEM of 6 mice per group for A-C and 8 mice per group for D-E. ^*^P<0.05; ^#^P<0.01; ^$^P<0.005.

Senescent cells accumulate in rodent and human WAT with advancing age [28]. Inflammatory mediators produced by senescent cells may contribute to age-related declines in WAT lipid storage. We tested if GH-related alterations in WAT lipid storage and differentiation capacity are associated with senescent cell burden. Snell dwarfs demonstrated a downward trend (P=0.088; Figure 3A) in ING WAT expression of p16^Ink4a^, a regulator of cellular senescence onset and maintenance [37, 38]. GHR-/- mice had significantly reduced ING WAT p16^Ink4a^ expression (P=0.012; Figure 3B). Since senescence only occurs in a small subset of normally replicating cells in any given tissue, changes in senescence-associated mRNA levels may be difficult to detect despite a substantial increase in senescent cell burden. Therefore, we counted senescent cells by assaying senescence-associated β-galactosidase positivity (SA-βgal^+^) as a function of total cell number. Senescent cell burden by this measure was lower in 18 month old Snell dwarf and GHR-/- mice than age-matched controls. Snell dwarf mice displayed a moderate genotype effect (P=0.058; Figure 4A) with significant reductions in ING, perirenal (PERI), and mesenteric (MES) depots. GHR-/- mice demonstrated a robust genotype difference in WAT senescent cell burden *vs*. controls (P<0.001; Figure 4B), with significant differences in all individual depots analyzed except MES WAT.

Analyses of bGH and chronically GH-injected mice support a link between cellular senescence and GH activity. Mice with increased GH levels by either method had significantly more senescent cell accumulation compared to their respective controls (P=0.002 and P=0.021, respectively; Figure 4C-D). bGH animals displayed significant differences in all depots with the exception of periovarian (POV) WAT. GH-injected animals had significant differences in subscapular (SCAP) and MES WAT. Increased GH was also associated with increased ING WAT p16^Ink4a^ and interleukin-6 (IL6) expression in bGH mice (P=0.010 and P=0.017, respectively; Figure 3C). No differences were found in these parameters in chronically GH-injected animals in ING WAT (Figure 3D), perhaps due to the low frequency of senescent cells as a function of total cell number in adipose tissue. The magnitude of transcriptional changes and senescent cell accumulation observed in bGH and GH-injected mice were similar to that of 24-month old mice (Figures 3E & 4E), despite the animals being much younger (14- and 5-months, respectively).

## DISCUSSION

Aging is a leading risk for the development of chronic diseases [39]. Redistribution of lipid from extra- to intra-peritoneal WAT depots occurs during aging with ectopic lipid accumulation [40]. These phenomena are associated with numerous age-related metabolic disorders, including low-grade inflammation, insulin resistance, nonalcoholic fatty liver disease, and type 2 diabetes mellitus [41]. Age-related metabolic dysfunction may be a consequence of declines in subcutaneous preadipocyte functional capacity, potentially due to increased inflammation and senescent cell accumulation [28, 42]. We previously found that eliminating senescent cells from progeroid mice extends healthspan, partially reverses age-related lipodystrophy, and delays aging-associated disorders [33]. Our current work suggests that GH activity could have a role in mediating the complicated interplay between the aforementioned age-related processes. Furthermore, mice with tempered GH action have enhanced health and lifespan as well as decreased age-related lipodystrophy. Thus, GH action, age-related WAT dysfunction, and senescent cell accumulation appear to be connected.

Subcutaneous WAT is specialized to store lipid as a long-term energy reserve. Sequestration of lipid in extra-peritoneal WAT depots may confer metabolic protection by preventing lipotoxicity and associated metabolic complications [42]. To investigate the connection between lipid deposition and aging, we analyzed WAT distribution in long-lived mice with diminished GH activity. We found that Ames dwarf, Snell dwarf, and GHR-/- mice all had higher extra-/intra-peritoneal WAT ratios than age-matched controls at 18 months of age. Consistent with this, others have shown a preferential expansion of subcutaneous WAT in GHR-/- mice [35, 36] and reduced epididymal WAT triglyceride accumulation in Ames dwarfs [34]. We also found that GHR-/- mice are protected from age-related accumulation of hepatic triglycerides. Others were not able to detect statistically significant increases in hepatic triglycerides in these mice [36], perhaps because of sample size, animal ages, or assay methods. Nevertheless, decreased age-related redistribution of lipid in mice with diminished GH activity supports the assertion that site-specific lipid deposition is linked with healthspan and longevity. It also suggests that endocrine actions of GH, and potentially IGF-1, may adversely affect preadipocyte function in mid- to late-life. Direct action of GH on primary rat preadipocytes is known to promote proliferation and inhibit differentiation in culture [43-45], whereas IGF-1 stimulates the inverse effects [46, 47]. We hypothesized that chronic exposure to GH and/or IGF-1 *in vivo* could blunt adipogenesis with advancing age. If true, ablation of GH-activity should curtail age-related declines in preadipocyte differentiation and provide a potential explanation for the preservation of extra-peritoneal WAT in the aforementioned models.

Preadipocyte differentiation is partially regulated through the adipogenic transcription factors, PPARγ and C/EBPα [48]. Both expression and activity of PPARγ and C/EBPα are reduced in an age-dependent manner [22, 23, 49]. To test the hypothesis that GH exposure inhibits preadipocyte differentiation in mid- to late-life, we assessed differentiation capacity of primary subcutaneous preadipocytes from GHR-/- and control mice at 20 months of age. We discovered that cells from GHR-/- mice had protection from age-related declines in adipogenesis. Moreover, PPARy and C/EBPα expression was higher in cells from GHR-/- mice than age-matched controls. Differentiation capacity of primary subcutaneous preadipocytes is similar in 4 month old GHR-/- and wild-type controls [50]. This suggests that long-term reductions in GH action prevent age-related declines in adipogenesis, as opposed to continuously enhancing adipogenesis throughout the lifespan independently of aging.

We speculate that reduced inflammation that is consistently observed in mice with decreased GH activity contributes to preserved adipogenesis [51]. Senescent cell accumulation may play a role due to the pro-inflammatory secretory profile of these cells. Senescent cells accumulate in WAT with advancing age [28]. Therefore, we hypothesized that preservation of adipogenesis in mice with reduced GH signaling may be associated with a reduction in senescent cell burden. Our suspicion was confirmed when Snell dwarf and GHR-/- mice were found to have fewer senescent cells in WAT than age-matched controls. We also found that Ames dwarf mice had reduced SA-βgal^+^ in ING WAT when compared to age-matched non-mutant littermates (data not shown). Conversely, bGH and chronically GH-injected animals were found to have more WAT senescent cell accumulation than their respective controls. Accumulation of SA-βgal^+^ cells was roughly mirrored by changes in p16^Ink4a^ transcription, which is usually increased in senescent cells [38]. Interestingly, the magnitude of senescent cell accumulation in younger bGH and chronically GH-injected mice was similar to that of 24 month old wild-type mice, reinforcing the contention that the premature aging phenotype observed in these models of GH excess could be related to senescent cell accumulation. This contention is supported by our previous work, in which the removal of senescent cells delays age-related lipoatrophy and enhances healthspan [33].

**Figure 5:**
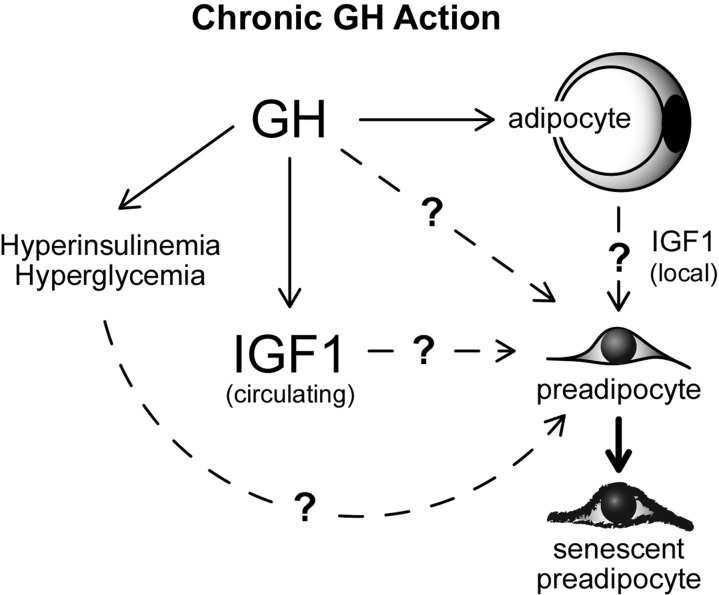
Speculative model of mechanisms contributing to GH-related WAT dysfunction with aging. Potential links between GH, IGF1, glucose, insulin and cellular senescence are indicated.

Our results demonstrate an association between GH activity, age-related WAT dysfunction, and WAT senescent cell accumulation in mice. However, it is currently unclear whether our findings are directly attributable to GH action in WAT. It is plausible that GH induces WAT cellular senescence through accelerating quiescent preadipocytes into a state of senescence (Figure 5); a process known as geroconversion [52, 53]. However, alterations in GH action also have profound effects on circulating levels of IGF-1, insulin, and glucose in every model we examined [10, 13]. Each of these variables is known to modulate mammalian target of rapamycin (mTOR) activity, which is essential for geroconversion and cellular senescence onset [54-60]. Indeed, previous work in culture has revealed that hyperglycemia and IGF-1 can induce cellular senescence in fibroblasts [61-63]. Hyperinsulinemia has also been reported to increase cellular senescence in cultured endothelial cells [64]. The role of IGF-1, insulin, and glucose in preadipocyte geroconversion remains to be investigated, although they all may contribute (Figure 5). Another unresolved possibility is that GH action in early-life may predispose various organ systems to dysfunction in mid- to late-life, thereby contributing to WAT dysfunction and senescent cell accumulation with aging. Interestingly, recent reports indicate that early-life GH replacement partially abrogates longevity in Ames dwarf mice [65, 66]. It is currently unknown if a critical window of GH action is responsible for effects on longevity.

In summary, long-lived GH-deficient and -resistant mice have reduced age-related lipid redistribution associated with reduced hepatic triglyceride accumulation, at least in GHR-/- mice. One mechanism potentially contributing to delayed age-related lipoatrophy, lipotoxicity, and dysfunction in GH-related mutants could be preservation of preadipocyte differentiation with advancing age. GH activity predicts senescent cell accumulation in WAT. Targeting links between GH action on the one hand and impaired adipogenesis, adipose tissue cellular senescence, and inflammation on the other may prove to be a path towards delaying age-related adipose and metabolic dysfunction and potentially enhancing healthspan.

## MATERIALS AND METHODS

### Animals and tissue collection

Eighteen-month old female Ames dwarf and non-mutant littermates were obtained from a breeding colony maintained by the Bartke laboratory at Southern Illinois University (SIU). Ames mice are on a heterogeneous genetic background. Eighteen-month old female Snell dwarf and non-mutant littermates were obtained from a breeding colony maintained by the Miller laboratory at University of Michigan. Snell mice are on a heterogeneous genetic background. Eighteen-month old female GHR-/- and wild-type littermates were obtained from a breeding colony maintained by the Kopchick laboratory at Ohio University (OU). An additional group of 20-month old GHR-/- and wild-type littermates were later obtained from the Kopchick laboratory for primary culture studies. Ten-month old female bGH [35] and wild-type controls were obtained from a breeding colony maintained by the Kopchick laboratory at OU. Both GHR-/- and bGH mice and their controls are on a C57BL/6 genetic background. Mice for porcine GH and saline treatment were obtained from the NIA colony (C57BL/6; Charles River Laboratories, Wilmington, MA) at approximately 14-months of age. Subcutaneous injections began at 15-months of age and were performed twice daily at a dose of 3 μg/g of bodyweight for four months. All injections were performed by members of the Bartke laboratory at SIU. Three-month and 24-month old female mice (C57BL/6) were purchased from the NIA colony and were analyzed at Mayo Clinic. All mice were housed 4-5 animals/cage at 22 ± 0.5°C on a 12:12-hour light-dark cycle at their respective institutions. Throughout the studies, mice were provided with *ad libitum* access to standard laboratory chow and water. All mice were euthanized by CO_2_ and tissues were immediately excised. WAT depots including ING, SCAP, POV, PERI, and MES were immediately excised and weighed prior to being prepared for SA-βgal^+^ cell counting and transcriptional analyses. Liver and other remaining tissues were flash-frozen in liquid nitrogen and stored at -80°C for future analyses. Extra-/intra-peritoneal WAT ratios were calculated by dividing the sum of the weights of ING, SCAP, and PERI by the sum of the weights of POV and MES. All procedures were approved by the Institutional Animal Care and Use Committees of each respective institution.

### Hepatic triglycerides

Triglyceride content within mouse liver was analyzed as previously described [67]. In brief, liver was homogenized in NETN lysis buffer supplemented with 5 mM NaF (Sigma-Aldrich, St. Louis, MO), 50 mM 2-glycerophosphate (Sigma-Aldrich), and protease inhibitor cocktail (Roche, Nutley, NJ). Lipid content was determined using Infinity Triglycerides Reagent (Thermo Fisher Scientific, Waltham, MA) according to the manufacturer's protocol.

### Cell culture

Primary ING preadipocytes were isolated from 20-month old female GHR-/- and wild-type littermates as previously described [68]. In brief, ING WAT was minced, digested with collagenase type-2 (Worthington Biochemical Corp., Lakewood, NJ, 1 mg/ml HBBS, Gibco,Grand Island, NY), filtered through a 100 μm nylon mesh, and centrifuged at 1000 x g for 10 minutes. The pellets were resuspended in αMEM containing 10% NBCS (Gibco) and 1% penicillin-streptomycin (Gibco). Cells were then plated and maintained in a humidified incubator for 16 hours with 3% O_2_ and 5% CO_2_ before being washed, trypsinized, and replated at a density of 5x10^4^ cells/cm^2^. This procedure results in over 90% pure preadipocyte populations as determined by morphology and assay of markers by RT-PCR [21, 69]. Differentiation of preadipocyte cultures was induced by exposure to differentiation medium containing DMEM/F-12 (Gibco), 10% FBS (Gibco), 1 μg/ml bovine insulin (Sigma-Aldrich), 250 nM dexamethasone (Sigma-Aldrich), 0.5 mM IBMX (Sigma-Aldrich), and 2.5 μM rosiglitazone (GlaxoSmithKline, Philadelphia, PA). Cells were differentiated for 48 hours in a humidified incubator with 20% O_2_ and 5% CO_2_. Cultures were then visually analyzed for lipid droplet formation prior to being lysed for transcriptional analysis of adipogenesis markers.

### Senescence-associated γ-galactosidase

Cellular SA-βgal^+^ was assessed as previously described [33]. In brief, immediately following excision approximately 75 mg of each WAT depot were rinsed in PBS and subsequently fixed for 10 minutes in PBS containing 2% formaldehyde (Sigma-Aldrich) and 0.25% glutaraldehyde (Sigma-Aldrich). Following fixation, tissues were washed three times for 5 minutes in PBS prior to being incubated for 18 hours at 37°C in SA-βgal activity solution (pH 6.0). The enzymatic reaction was then stopped with ice-cold PBS and tissues were again washed three times for 5 minutes in PBS. Tissues were then incubated for 10 minutes in Hoescht 33342 solution (2.5 μg/ml, Life Technologies,Grand Island, NY) to stain nuclei. Tissues were then placed between two mounting slides and 8-10 images were taken from random fields using phase contrast and fluorescence settings (Nikon Eclipse T*i*, Melville, NY). SA-βgal^+^ cells in each field were then counted and normalized to the total number of nuclei in the same field by blinded reviewers. As a means to standardize across several experiments, SA-βgal^+^ cells within each mouse and depot were normalized as a percentage of the total number of senescent cells observed in their respective SA-βgal experiment.

### Real-time PCR

Total RNA was extracted from preadipocyte cultures and WAT using TRIzol (Life Technologies) according to the manufacturer's protocol. cDNA was generated using the SuperScript III First-Strand Synthesis System (Life Technologies). Real-time PCR was performed in a 7500 Fast Real Time PCR System using TaqMan Fast Universal PCR Master Mix and predesigned primers and probes from Applied Biosystems (Foster City, CA). Target gene expression was expressed as 2^-ΔΔCT^ by the comparative CT method [70] and normalized to the expression of TATA-binding protein (TBP).

### Statistical analyses

Unless otherwise noted, differences were analyzed by Student's t-tests. For SA-βgal^+^ data, mixed effects models were used to perform two-way ANOVA with interaction while accounting for the correlation between multiple observations per mouse. Akaike information criterion (AIC) was used to choose between two covariance structures: i) compound symmetry which assumes equal correlation between all depots and therefore minimizes the number of parameters to estimate, and ii) unstructured which allows for a different correlation between each pair of depots, resulting in a larger number of parameters to estimate. Compound symmetry was chosen for all experiments. For the Snell dwarf, GHR-/- and bGH experiments, a common covariance structure was assumed. For the Injected and Young *vs.* Old experiments, covariance matrices were estimated within genotype to allow for different variances. All data are presented as mean ± SEM with P<0.05 considered significantly different. GraphPad Prism 5 (La Jolla, CA) or SAS v9.0 (Cary, NC) were used for all statistical analyses.

## References

[R1] Bartke A (2003). Can growth hormone (GH) accelerate aging? Evidence from GH-transgenic mice. Neuroendocrinology.

[R2] Ayuk J, Sheppard MC (2008). Does acromegaly enhance mortality?. Rev Endocr Metab Disord.

[R3] Everitt AV, Seedsman NJ, Jones F (1980). The effects of hypophysectomy and continuous food restriction, begun at ages 70 and 400 days, on collagen aging, proteinuria, incidence of pathology and longevity in the male rat. Mech Ageing Dev.

[R4] Shimokawa I, Higami Y, Utsuyama M, Tuchiya T, Komatsu T, Chiba T, Yamaza H (2002). Life span extension by reduction in growth hormone-insulin-like growth factor-1 axis in a transgenic rat model. Am J Pathol.

[R5] Brown-Borg HM, Borg KE, Meliska CJ, Bartke A (1996). Dwarf mice and the ageing process. Nature.

[R6] Flurkey K, Papaconstantinou J, Miller RA, Harrison DE (2001). Lifespan extension and delayed immune and collagen aging in mutant mice with defects in growth hormone production. Proc Natl Acad Sci U S A.

[R7] Coschigano KT, Holland AN, Riders ME, List EO, Flyvbjerg A, Kopchick JJ (2003). Deletion, but not antagonism, of the mouse growth hormone receptor results in severely decreased body weights, insulin, and insulin-like growth factor I levels and increased life span. Endocrinology.

[R8] Guevara-Aguirre J, Balasubramanian P, Guevara-Aguirre M, Wei M, Madia F, Cheng CW, Hwang D, Martin-Montalvo A, Saavedra J, Ingles S, de Cabo R, Cohen P, Longo VD (2011). Growth hormone receptor deficiency is associated with a major reduction in pro-aging signaling, cancer, and diabetes in humans. Sci Transl Med.

[R9] Milman S, Atzmon G, Huffman DM, Wan J, Crandall JP, Cohen P, Barzilai N (2014). Low insulin-like growth factor-1 level predicts survival in humans with exceptional longevity. Aging Cell.

[R10] Bartke A, Sun LY, Longo V (2013). Somatotropic signaling: trade-offs between growth, reproductive development and longevity. Physiol Rev.

[R11] Andersen B, Pearse RV, Jenne K, Sornson M, Lin SC, Bartke A, Rosenfeld MG (1995). The Ames dwarf gene is required for Pit-1 gene activation. Dev Biol.

[R12] Li S, Crenshaw EB, Rawson EJ, Simmons DM, Swanson LW, Rosenfeld MG (1990). Dwarf locus mutants lacking three pituitary cell types result from mutations in the POU-domain gene pit-1. Nature.

[R13] Junnila RK, List EO, Berryman DE, Murrey JW, Kopchick JJ (2013). The GH/IGF-1 axis in ageing and longevity. Nat Rev Endocrinol.

[R14] List EO, Sackmann-Sala L, Berryman DE, Funk K, Kelder B, Gosney ES, Okada S, Ding J, Cruz-Topete D, Kopchick JJ (2011). Endocrine parameters and phenotypes of the growth hormone receptor gene disrupted (GHR-/-) mouse. Endocr Rev.

[R15] Palmer AJ, Chung MY, List EO, Walker J, Okada S, Kopchick JJ, Berryman DE (2009). Age-related changes in body composition of bovine growth hormone transgenic mice. Endocrinology.

[R16] Baker DJ, Jeganathan KB, Malureanu L, Perez-Terzic C, Terzic A, van Deursen JM (2006). Early aging-associated phenotypes in Bub3/Rae1 haploinsufficient mice. J Cell Biol.

[R17] DeNino WF, Tchernof A, Dionne IJ, Toth MJ, Ades PA, Sites CK, Poehlman ET (2001). Contribution of abdominal adiposity to age-related differences in insulin sensitivity and plasma lipids in healthy nonobese women. Diabetes Care.

[R18] Hughes VA, Roubenoff R, Wood M, Frontera WR, Evans WJ, Fiatarone Singh MA (2004). Anthropometric assessment of 10-y changes in body composition in the elderly. Am J Clin Nutr.

[R19] Djian P, Roncari AK, Hollenberg CH (1983). Influence of anatomic site and age on the replication and differentiation of rat adipocyte precursors in culture. J Clin Invest.

[R20] Kirkland JL, Hollenberg CH (1998). Inhibitors of preadipocyte replication: opportunities for the treatment of obesity. Prog Mol Subcell Biol.

[R21] Kirkland JL, Hollenberg CH, Gillon WS (1990). Age, anatomic site and the replication and differentiation of adipocyte precursors. Am J Physiol.

[R22] Schipper BM, Marra KG, Zhang W, Donnenberg AD, Rubin JP (2008). Regional anatomic and age effects on cell function of human adipose-derived stem cells. Ann Plast Surg.

[R23] Karagiannides I, Tchkonia T, Dobson DE, Steppan CM, Cummins P, Chan G, Salvatori K, Hadzopoulou-Cladaras M, Kirkland JL (2001). Altered expression of C/EBP family members results in decreased adipogenesis with aging. Am J Physiol Regul Integr Comp Physiol.

[R24] Karagiannides I, Thomou T, Tchkonia T, Pirtskhalava T, Kypreos KE, Cartwright A, Dalagiorgou G, Lash TL, Farmer SR, Timchenko NA, Kirkland JL (2006). Increased CUG triplet repeat-binding protein-1 predisposes to impaired adipogenesis with aging. J Biol Chem.

[R25] Tchkonia T, Pirtskhalava T, Thomou T, Cartwright MJ, Wise B, Karagiannides I, Shpilman A, Lash TL, Becherer JD, Kirkland JL (2007). Increased TNFalpha and CCAAT/enhancer-binding protein homologous protein with aging predispose preadipocytes to resist adipogenesis. Am J Physiol Endocrinol Metab.

[R26] Morin CL, Pagliassotti MJ, Windmiller D, Eckel RH (1997). Adipose tissue-derived tumor necrosis factor-alpha activity is elevated in older rats. J Gerontol A Biol Sci Med Sci.

[R27] Cartwright MJ, Schlauch K, Lenburg ME, Tchkonia T, Pirtskhalava T, Cartwright A, Thomou T, Kirkland JL (2010). Aging, depot origin, and preadipocyte gene expression. J Gerontol A Biol Sci Med Sci.

[R28] Tchkonia T, Morbeck DE, Von Zglinicki T, Van Deursen J, Lustgarten J, Scrable H, Khosla S, Jensen MD, Kirkland JL (2010). Fat tissue, aging, and cellular senescence. Aging Cell.

[R29] Coppe JP, Patil CK, Rodier F, Sun Y, Munoz DP, Goldstein J, Nelson PS, Desprez PY, Campisi J (2008). Senescence-associated secretory phenotypes reveal cell-nonautonomous functions of oncogenic RAS and the p53 tumor suppressor. PLoS Biol.

[R30] Freund A, Orjalo AV, Desprez PY, Campisi J (2010). Inflammatory networks during cellular senescence: causes and consequences. Trends Mol Med.

[R31] Parrinello S, Coppe JP, Krtolica A, Campisi J (2005). Stromal-epithelial interactions in aging and cancer: senescent fibroblasts alter epithelial cell differentiation. J Cell Sci.

[R32] Xue W, Zender L, Miething C, Dickins RA, Hernando E, Krizhanovsky V, Cordon-Cardo C, Lowe SW (2007). Senescence and tumour clearance is triggered by p53 restoration in murine liver carcinomas. Nature.

[R33] Baker DJ, Wijshake T, Tchkonia T, LeBrasseur NK, Childs BG, van de Sluis B, Kirkland JL, van Deursen JM (2011). Clearance of p16Ink4a-positive senescent cells delays ageing-associated disorders. Nature.

[R34] Wang Z, Al-Regaiey KA, Masternak MM, Bartke A (2006). Adipocytokines and lipid levels in Ames dwarf and calorie-restricted mice. J Gerontol A Biol Sci Med Sci.

[R35] Berryman DE, List EO, Coschigano KT, Behar K, Kim JK, Kopchick JJ (2004). Comparing adiposity profiles in three mouse models with altered GH signaling. Growth Horm IGF Res.

[R36] Berryman DE, List EO, Palmer AJ, Chung MY, Wright-Piekarski J, Lubbers E, O'Connor P, Okada S, Kopchick JJ (2010). Two-year body composition analyses of long-lived GHR null mice. J Gerontol A Biol Sci Med Sci.

[R37] Campisi J (2005). Senescent cells tumor suppression and organismal aging: good citizens, bad neighbors. Cell.

[R38] Jeyapalan JC, Sedivy JM (2008). Cellular senescence and organismal aging. Mech Ageing Dev.

[R39] Kirkland JL (2013). Translating advances from the basic biology of aging into clinical application. Exp Gerontol.

[R40] Kuk JL, Saunders TJ, Davidson LE, Ross R (2009). Age-related changes in total and regional fat distribution. Ageing Res Rev.

[R41] Guo SS, Zeller C, Chumlea WC, Siervogel RM (1999). Aging, body composition, and lifestyle: the Fels Longitudinal Study. Am J Clin Nutr.

[R42] Tchkonia T, Thomou T, Zhu Y, Karagiannides I, Pothoulakis C, Jensen MD, Kirkland JL (2013). Mechanisms and Metabolic Implications of Regional Differences among Fat Depots. Cell Metab.

[R43] Hauner H, Loffler G (1987). Adipose tissue development: the role of precursor cells and adipogenic factors. Part I: Adipose tissue development and the role of precursor cells. Klin Wochenschr.

[R44] Loffler G, Hauner H (1987). Adipose tissue development: the role of precursor cells and adipogenic factors. Part II: The regulation of the adipogenic conversion by hormones and serum factors. Klin Wochenschr.

[R45] Wabitsch M, Heinze E, Hauner H, Shymko RM, Teller WM, De Meyts P, Ilondo MM (1996). Biological effects of human growth hormone in rat adipocyte precursor cells and newly differentiated adipocytes in primary culture. Metabolism.

[R46] Wabitsch M, Hauner H, Heinze E, Teller WM (1995). The role of growth hormone/insulin-like growth factors in adipocyte differentiation. Metabolism.

[R47] Deslex S, Negrel R, Ailhaud G (1987). Development of a chemically defined serum-free medium for differentiation of rat adipose precursor cells. Exp Cell Res.

[R48] Rosen ED, MacDougald OA (2006). Adipocyte differentiation from the inside out. Nat Rev Mol Cell Biol.

[R49] Hotta K, Bodkin NL, Gustafson TA, Yoshioka S, Ortmeyer HK, Hansen BC (1999). Age-related adipose tissue mRNA expression of ADD1/SREBP1, PPARgamma, lipoprotein lipase, and GLUT4 glucose transporter in rhesus monkeys. J Gerontol A Biol Sci Med Sci.

[R50] Flint DJ, Binart N, Boumard S, Kopchick JJ, Kelly P (2006). Developmental aspects of adipose tissue in GH receptor and prolactin receptor gene disrupted mice: site-specific effects upon proliferation, differentiation and hormone sensitivity. J Endocrinol.

[R51] Masternak MM, Bartke A (2012). Growth hormone inflammation and aging. Pathobiol Aging Age Relat Dis.

[R52] Blagosklonny MV (2012). Cell cycle arrest is not yet senescence which is not just cell cycle arrest: terminology for TOR-driven aging. Aging (Milano).

[R53] Sousa-Victor P, Gutarra S, Garcia-Prat L, Rodriguez-Ubreva J, Ortet L, Ruiz-Bonilla V, Jardi M, Ballestar E, Gonzalez S, Serrano AL, Perdiguero E, Munoz-Canoves P (2014). Geriatric muscle stem cells switch reversible quiescence into senescence. Nature.

[R54] Leontieva OV, Demidenko ZN, Blagosklonny MV (2014). Contact inhibition and high cell density deactivate the mammalian target of rapamycin pathway, thus suppressing the senescence program. Proc Natl Acad Sci U S A.

[R55] Leontieva OV, Demidenko ZN, Blagosklonny MV (2013). S6K in geroconversion. Cell Cycle.

[R56] Demidenko ZN, Zubova SG, Bukreeva EI, Pospelov VA, Pospelova TV, Blagosklonny MV (2009). Rapamycin decelerates cellular senescence. Cell Cycle.

[R57] Leontieva OV, Demidenko ZN, Blagosklonny MV (2013). MEK drives cyclin D1 hyperelevation during geroconversion. Cell Death Differ.

[R58] Iglesias-Bartolome R, Gutkind JS (2012). Exploiting the mTOR paradox for disease prevention. Oncotarget.

[R59] Luo Y, Li L, Zou P, Wang J, Shao L, Zhou D, Liu L (2014). Rapamycin enhances long-term hematopoietic reconstitution of ex vivo expanded mouse hematopoietic stem cells by inhibiting senescence. Transplantation.

[R60] Iglesias-Bartolome R, Patel V, Cotrim A, Leelahavanichkul K, Molinolo AA, Mitchell JB, Gutkind JS (2012). mTOR inhibition prevents epithelial stem cell senescence and protects from radiation-induced mucositis. Cell Stem Cell.

[R61] Blazer S, Khankin E, Segev Y, Ofir R, Yalon-Hacohen M, Kra-Oz Z, Gottfried Y, Larisch S, Skorecki KL (2002). High glucose-induced replicative senescence: point of no return and effect of telomerase. Biochem Biophys Res Commun.

[R62] Handayaningsih AE, Takahashi M, Fukuoka H, Iguchi G, Nishizawa H, Yamamoto M, Suda K, Takahashi Y (2012). IGF-I enhances cellular senescence via the reactive oxygen species-p53 pathway. Biochem Biophys Res Commun.

[R63] Tran D, Bergholz J, Zhang H, He H, Wang Y, Zhang Y, Li Q, Kirkland JL, Xiao Z-X (2014). Insulin-like growth factor-1 regulates the SIRT1-p53 pathway in cellular senescence. Aging Cell.

[R64] Matsui-Hirai H, Hayashi T, Yamamoto S, Ina K, Maeda M, Kotani H, Iguchi A, Ignarro LJ, Hattori Y (2011). Dose-dependent modulatory effects of insulin on glucose-induced endothelial senescence in vitro and in vivo: a relationship between telomeres and nitric oxide. J Pharmacol Exp Ther.

[R65] Masternak MM, Panici JA, Wang F, Wang Z, Spong A (2010). The effects of growth hormone (GH) treatment on GH and insulin/IGF-1 signaling in long-lived Ames dwarf mice. J Gerontol A Biol Sci Med Sci.

[R66] Panici JA, Harper JM, Miller RA, Bartke A, Spong A, Masternak MM (2010). Early life growth hormone treatment shortens longevity and decreases cellular stress resistance in long-lived mutant mice. Faseb J.

[R67] Escande C, Chini CC, Nin V, Dykhouse KM, Novak CM, Levine J, van Deursen J, Gores GJ, Chen J, Lou Z, Chini EN (2010). Deleted in breast cancer-1 regulates SIRT1 activity and contributes to high-fat diet-induced liver steatosis in mice. J Clin Invest.

[R68] Kirkland JL, Hollenberg CH, Gillon WS (1996). Effects of fat depot site on differentiation-dependent gene expression in rat preadipocytes. Int J Obes Relat Metab Disord.

[R69] Wang H, Kirkland JL, Hollenberg CH (1989). Varying capacities for replication of rat adipocyte precursor clones and adipose tissue growth. J Clin Invest.

[R70] Livak KJ, Schmittgen TD (2001). Analysis of relative gene expression data using real-time quantitative PCR and the 2(-Delta Delta C(T)) Method. Methods.

